# Health Status Stability of Patients in a Medical Rehabilitation Program: What Are the Roles of Time, Physical Fitness Level, and Self-efficacy?

**DOI:** 10.1007/s12529-021-10046-6

**Published:** 2021-12-23

**Authors:** Qianqian Ju, Yiqun Gan, Robin Rinn, Yanping Duan, Sonia Lippke

**Affiliations:** 1 School of Psychological and Cognitive Sciences, Beijing Key Laboratory of Behavior and Mental Health, Peking University, Beijing, China; 2 University of Würzburg, Wurzburg, Germany; 3 Hong Kong Baptist University, Kowloon, Hong Kong; 4 Jacobs University Bremen, Bremen, Germany

**Keywords:** Latent growth curve model, Mental health, Physical fitness, Self-efficacy, Physical health

## Abstract

**Background:**

Individuals’ physical and mental health, as well as their chances of returning to work after their ability to work is damaged, can be addressed by medical rehabilitation.

**Aim:**

This study investigated the developmental trends of mental and physical health among patients in medical rehabilitation and the roles of self-efficacy and physical fitness in the development of mental and physical health.

**Design:**

A longitudinal design that included four time-point measurements across 15 months.

**Setting:**

A medical rehabilitation center in Germany.

**Population:**

Participants included 201 patients who were recruited from a medical rehabilitation center.

**Methods:**

To objectively measure physical fitness (lung functioning), oxygen reabsorption at anaerobic threshold (VO2AT) was used, along with several self-report scales.

**Results:**

We found a nonlinear change in mental health among medical rehabilitation patients. The results underscored the importance of medical rehabilitation for patients’ mental health over time. In addition, patients’ physical health was stable over time. The initial level of physical fitness (VO2AT) positively predicted their mental health and kept the trend more stable. Self-efficacy appeared to have a positive relationship with mental health after rehabilitation treatment.

**Conclusions:**

This study revealed a nonlinear change in mental health among medical rehabilitation patients. Self-efficacy was positively related to mental health, and the initial level of physical fitness positively predicted the level of mental health after rehabilitation treatment.

**Clinical Rehabilitation:**

More attention could be given to physical capacity and self-efficacy for improving and maintaining rehabilitants’ mental health.

**Supplementary Information:**

The online version contains supplementary material available at 10.1007/s12529-021-10046-6.

## Introduction

The modern world brings new challenges to the health of workers and employees, and sedentary work and lack of physical activity are just two risk factors affecting workers’ physical and mental health [1–3]. Facilitating employee exercise can help them remain healthy [4] or become healthy again after illness [5]. If an employee’s health is affected by illness or an accident that leads to a reduced ability to work, many countries such as Germany offer medical rehabilitation to improve health, functionality, ability to work and also social participation. Rehabilitation treatment includes physical activity, psychological counseling, and the improvement of physical fitness as integral parts. To explore the role of rehabilitation in patients’ physical and mental health, functionality, capacity for social participation, and the interrelating factors of medical rehabilitation, this study recorded the trajectories of mental and physical health during and after rehabilitation using a 15-month longitudinal design. More specifically, a combination of objective and subjective measurements was used to examine the predicted role of physical fitness in mental and physical health, and to explore the relationships between self-efficacy and mental and physical health.

## Medical Rehabilitation to Improve Health

Medical rehabilitation aims at partial or complete (re-)integration into working life [6, 7]. Based on the fundamental principle of the German pension fund, the insurance of such medical rehabilitation for all insured people, described as Rehabilitation, has priority over a pension (retrieved from https://www.deutsche-rentenversicherung.de/DRV/EN/Leistungen/leistungen_node.html). In Germany, medical rehabilitation typically lasts 3 weeks and is mainly delivered within specialized rehabilitation clinics [7, 8]. It is provided only if people are not able to work or are at risk for long-term reduced social participation.

Rehabilitation includes work-based exercises [e.g., shoulder and neck exercises, 9], and it has been shown to improve mental and physical health [10–12]. Bethge [13] determined the positive role of medical rehabilitation including exercises to improve ability to work among patients with chronic back pain through an elaborate cohort study. Other studies also found positive functions of rehabilitation, not only for ability to work [14, 15], but also for patients’ well-being [16], health status [17], and living conditions [18]. However, group-level analysis (such as ANOVA) ignores individual differences, especially in the process of long-term development, and group-level analysis alone cannot determine whether all individuals follow the same trend. If individual-level analysis could be conducted, ascertaining whether individual development follows the same trajectory and identifying the key time points in the individual development trends, then, the required support could be provided at the key time point, and the intervention effect can be more efficiently maximized [9].

To explore the development of mental and physical health of participants individually, and examine whether all participants follow the same trend together, a 15-month longitudinal study with four time points was conducted. ANOVA was conducted to investigate the development of patients’ physical and mental health at the group level, while the latent growth curve model (LGCM) was used to explore the trend for participants individually.

## Mental Health, Physical Health, and Returning to Work

There are two important goals that should be achieved through rehabilitation before returning to work: first, mental health [7]; second, physical health [19].

Previous cohort studies, such as Tengland [20], have shown that patients with average ability to work have better physical and mental health and a lower number of absences due to sickness. Therefore, mental health is a crucial factor for rehabilitants as poor mental health increases employees’ mental stressors and reduces their subjective ability to work [21, 22].

Physical health is another essential factor for rehabilitation patients to be able to return to work [23]. As relating to physical work capacity, physical health is highly connected with mental health and return to work after rehabilitation [24, 25] and lays the basis for mental well-being and the ability to buffer the effects of stressors [19].

Subjective measurements are widely used to assess physical health [26], while objective measurements such as physical capacity are also important to consider as they are less likely to underlie social desirability or motivational influences, which typically play a role in self-report measurements. Furthermore, it is likely that such measurements offer unique contributions to an understanding of rehabilitation processes, which may facilitate or hinder successful medical rehabilitation. Thus, the present study used a relatively novel objective measurement in psychology, namely, lung functioning or oxygen reabsorption at anaerobic threshold (VO2AT).

At present, little is known about the developmental tendencies towards mental and physical health among employees undergoing medical rehabilitation. Specifically, it is still unknown whether the patients’ mental and physical health changes over time during and after medical rehabilitation. Therefore, this study used a longitudinal design and subjective and objective indicators to directly reflect the developmental trajectories of mental and physical health in rehabilitants. For this purpose, and on the basis of the literature reviewed, the following hypotheses were derived:

### Hypothesis 1

Patients’ mental health improves over time, and therefore, we expect an improvement in mental health at T2, T3, and T4 compared to T1.

### Hypothesis 2

Patients’ physical health improves over time.

## Physical Fitness, Physical Capacity, and VO2AT

In addition to the self-reports of ability to work and health, physical capacity, which includes cardiovascular fitness, muscular strength, muscular endurance, flexibility, and body composition [27], is important for the success of a rehabilitation [28]. As an objective measure of physical fitness, VO2AT is one index of cardiopulmonary capacity, which falls into the domain of cardiovascular fitness. VO2AT signifies the oxygen consumption of the anaerobic threshold reflecting an individual’s physical fitness in many areas [18, 23, 29, 30]. Physical fitness plays an important role in maintaining physical health by alleviating clustered cardiometabolic risk [31], total and abdominal adiposity, traditional and emerging cardiovascular disease risk factors, and others [32] and has also been found to be associated with mental health [33, 34]. This study aimed to test the role of VO2AT on the developmental tendencies of mental and physical health, using the following hypotheses:

### Hypothesis 3

The level of initial physical fitness (T1) is a positive predictor of improvement in mental health over time.

### Hypothesis 4

The level of initial physical fitness (T1) is a positive predictor of improvement of physical health over time.

## The Role of Self-Efficacy and the Compensatory Carry-Over Action Model

The compensatory carry-over action model [CCAM, 35] explains that health-related behaviors and social-cognitive determinants such as self-efficacy are interconnected. That is, within this perspective, a persons’ self-efficacy and well-being develop from the experience that a person is able to be physically active [36–39] and vice versa. Self-efficacy expectation is defined as the belief by an individual that they are able to successfully perform a specific behavior [40, 41], and whether this behavior is expected to generate specific outcomes is conceptualized in response-outcome expectations. In the case of strong outcome expectations (i.e., a person is convinced that a behavior leads to a desired outcome), self-efficacy expectation is important as it includes the belief that individuals can successfully initiate and maintain their behavior to ultimately produce the outcome [42]. Research has impressively shown in different meta-analyses that self-efficacy was the main driver of different behaviors and health-related outcomes [43, 44], health-related behavior [45], and quality of life [46].

## Long-Term Role of Rehabilitation Treatment

The long-term interrelation of rehabilitation treatment for the development of health behaviors has been explored in several studies [47–49]. Boesen [50] found inpatient multidisciplinary rehabilitation led to long-lasting improvement in health-related quality of life for multiple sclerosis patients. Pietila-Holmner, Enthoven [51] found multimodal rehabilitation programmers in primary care were beneficial for pain, physical and emotional functioning, coping, and health-related quality of life at 1-year follow-up for patients with chronic pain.

To sum up, self-efficacy plays an important role in improving mental health and physical activity, but little is known about how the changes in health experiences are interrelated with self-efficacy and physical fitness, and at what time point self-efficacy plays a role in the development of mental/physical health. Therefore, another aim was to test whether self-efficacy has a long-term interrelation with mental and physical health of patients due to the interaction of physical fitness.

### Hypothesis 5

Self-efficacy is positively associated with mental health at each time point.

### Hypothesis 6

Self-efficacy is positively associated with physical health at each time point.

## The Present Study

To investigate the developmental trends of mental and physical health among medical rehabilitation patients and explore the role of physical fitness and self-efficacy, this study used the LGCM with a longitudinal design. The LGCM is a suitable method for exploring the tendency and the interrelating factor of rehabilitation treatment with health-related behaviors in this study [52]. For our hypotheses, we aimed to explore individual changes over time. The advantage of an LGCM over repeated measures ANCOVA is that it provides a model fit for both the intraindividual (within-person) and interindividual (between-person) changes over time. It could also examine whether all individuals follow a similar development trend [53, 54]. Previous studies have used the LGCM to explore the long-term development of health behaviors, such as physical and psychological health [55], exercise behaviors [56], and quality of life [57].

## Method

### Participants

In this study, 201 participants were recruited from a medical rehabilitation center in Germany and took part in a VO2AT test. All participants were rehabilitation patients. Of the recruited participants, 200 completed the questionnaire and provided useful data. One individual was excluded due to lack of questionnaire data; therefore, 200 participants constituted the final sample. A summary of the demographic characteristics of the participants is shown in Table 1.

**Table 1:** Demographic data at time 1

		*n*	*Percent*	*M*	*SD*
Age (range: 29–63 years)				52.16	6.83
Sex	Male	47	23.50%		
	Female	153	76.50%		
Working state	Unable to work	69	34.50%		
	Unemployed	15	7.50%		
	Working	116	58.00%		
Orthopedic diagnoses*	Spine	151	75.50%		
	Joints	49	24.50%		
	number of orthopedic diagnoses			2.72	1.05

Data reported here are from T1

^*^Participants have orthopedic diagnoses (*Range: 1-10, Mode* = 2, *Median* = 3)

### Procedure

All participants were fully informed about the study and completed an informed consent form before taking part in this study. All procedures were in accordance with the ethical standards of the responsible committee on human experimentation (institutional and national) and with the Helsinki Declaration of 1975, as revised in 2000. The study was approved by the Ethics Committee of the German Psychological Society (DGPs).

At T1, participants were recruited during rehabilitation, participated in the VO2AT test, and completed paper–pencil questionnaires as described below. The following measurement time points (T2, T3, T4) took place after rehabilitation and self-report data were collected by computer-assisted telephone interviewing (CATI). The interviewers were student assistants supervised by an experienced researcher. Dropouts were due to lack of interest or inability to stay in contact. The demographic variables and VO2AT were measured only at baseline (T1), while questions regarding health and self-efficacy were measured at each time point. T2 was 7 months after the beginning of rehabilitation, T3 was 12 months, and T4 was 15 months after the beginning of rehabilitation. All data were merged by the participants’ unique number to ensure participant anonymity. The overview of the data collection process is shown in Table 2.

**Table 2:** Overview of the data collection

Measurement points	VO2AT test	T1	T2	T3	T4
Months after T1	0	0	7	12	15
Measurement method	Spirometric test at rehabilitation clinic	Paper-pencil questionnaire	Telephone interview (CATI)	Telephone interview (CATI)	Telephone interview (CATI)
Instruments	/	SE, SF-12	SE, SF-12	SE, SF-12	SE, SF-12
*N*	201	200	139*	103*	73*

*SE* self-efficacy, *SF-12* SF-12 health survey, *T1* ~ *T4* Time1 ~ Time4

^*^Means the missing data has been imputed with the procedure of *k*-nearest neighbor imputation

### Measures

#### SF-12 Health Survey (SF-12)

The SF-12 is a short version of the SF-36 that includes a mental health component summary (MCS) and a physical health component summary (PCS). It reflects at least 90% measurements of the SF-36 [58, 59]. An example item is “In general, would you say your health is:” and the responses include excellent, very good, good, fair, and poor. Considering that calculating the total score of the SF-12 is not a simple sum, retest reliability was adopted here (as shown in Table 3 with bold font). The test–retest reliability coefficients for the MCS and PCS were significant (0.31–0.55), except for the values between T1 and T2–T4 for the MCS (0.02, 0.07, and 0.09), and between T1 and T2 for the PCS (0.07). The possible reason for these non-significant coefficients might be that, between T1 and T2, the rehabilitation worked, leading to the score changes measured by the SF-12 (as shown in the ANOVA results).

**Table 3:** Descriptive statistics and partial correlations among study variables

	Value	*Mean*	*SD*	1	2	3	4	5	6	7	8	9	10	11	12	13
1	VO2AT	15.32	3.34	1.00												
2	MCS_T1	44.09	10.00	.21**	1.00											
3	MCS_T2	53.89	9.03	− .05	.00	1.00										
4	MCS_T3	56.38	9.31	− .02	.05	.42***	1.00									
5	MCS_T4	53.26	8.16	− .03	.07	.31***	.40**	1.00								
6	PCS_T1	38.25	6.79	.08	− .23**	− .02	− .04	.00	1.00							
7	PCS_T2	39.05	7.49	.41***	.18*	− .11	− .08	.12	**.08**	1.00						
8	PCS_T3	38.78	6.56	.21**	.03	− .02	− .24**	− .03	.17*	.55***	1.00					
9	PCS_T4	37.71	6.37	.18**	.10	− .01	− .16*	− .10	.16*	.25***	.39***	1.00				
10	SE_T1	3.10	0.59	.06	.03	− .04	− .02	− .02	− .16*	.01	.00	− .03	1.00			
11	SE_T2	2.51	1.79	.04	.06	− .06	− .15*	− .01	− .08	.16*	.15*	.14	.01	1.00		
12	SE_T3	3.64	0.64	.07	.00	.17*	.50***	.07	.07	− .05	− .03	.02	.00	− .06	1.00	
13	SE_T4	3.81	0.45	− 0.01	.00	− .02	.30***	.16*	− .01	.11	.06	− .09	.07	.05	.32***	1.00

The results in bold indicate the test–retest reliabilities for MCS and PCS from T1 to T4

*MCS* mental health component summary, *PCS* physical health component summary, *SE* self-efficacy, *T1* ~ *T4* Time1 ~ Time4

^*^p < .050

^**^p < .010

#### Self-efficacy

Self-efficacy for health was assessed using the health action process approach (HAPA) model adapted from Schwarzer [60] and was measured by one item: “I am sure that I can lead a healthy life.” A 4-point Likert scale was used, where 1 = “not true,” 2 = “hardly true,” 3 = “rather true,” and 4 = “exactly true.”

##### VO2AT

was measured by a medical practitioner who specialized in cardiology and reflects the fitness capacity of the participants. Participants were asked to take part in a physical stress test (spinning) under constant physical exertion. During this time, spirometric data were collected by medical personnel. The VO2AT data provided information about the oxygen reabsorption of participants in percentages form: a higher VO2AT value means that participants are able to reabsorb more oxygen. Oxygen reabsorption is influenced by smoking, sex, and physical activity level [61]. VO2AT was only measured at T1.

## Statistical Analyses

Based on the functions of different statistical software, R 2.70 was used for imputation of missing values, IBM SPSS version 24.0 was used for dropout and descriptive analysis and ANCOVA analysis, and MPLUS version 7.2 was used to conduct LGCMs.

First, for the missing data, the patient and doctor’s joint decision about whether the patient can work again after rehabilitation was imputed on the basis of sex and age with the procedure of *k-*nearest neighbor imputation implemented in R [62]. Subsequently, all the missing data were imputed on the basis of sex, age, and the joint decision of patient and doctor with the same algorithm.^1^

Second, the descriptive statistics of each variable were calculated, and the Pearson correlation coefficients (*r*) between each pair of variables were analyzed. To explore the effectiveness of the intervention at the group level, ANOVA was conducted to compare the mental and physical health for four time points. Moreover, to explore the individual changes in the developmental tendencies for mental and physical health over time, unconditional linear and quadratic LGCMs were built. Furthermore, conditional LGCMs with VO2AT and self-efficacy for health were built to reflect their roles in the changes in mental and physical health. Insomuch that VO2AT changed little over a long time [63] and was only measured at T1, the VO2AT was considered the time-invariant covariate for the LGCM, while self-efficacy was a time-variant variable with four measurements.

For model fit of LGCM, the *χ*^2^ distribution, the root mean square error of approximation (RMSEA), the comparative fit index (CFI), and standardized root mean square residual (SRMR) were assessed. A RMSEA from 0.10 to 0.08 indicated a moderate model fit, and a RMSEA value lower than 0.08 indicated a good model fit; a CFI between 0.90 and 0.94 was considered an acceptable model fit, and a CFI value above 0.95 indicated an excellent model fit; an SRMR value below 0.08 was considered a good model fit [64, 65].

## Results

### Dropout and Descriptive Analysis

Attrition analyses revealed that, at T1, participants who dropped out at T4 did not differ from participants who were tested at T4. Thus, no differences were found in SF-12 and self-efficacy scores (all *t*_[199]_ < 1.11, all *ps* > 0.050) as well as VO2AT. There were also no differences in sex *χ*^2^(1) = 0.01, *p* > 0.56 (one-tailed). However, the participants who dropped out were slightly younger than those who took part at T4 (*t*_[199]_ =  − 2.46, *p* = 0.023; *M*_dropout_ = 51.27, *SD* = 6.76; *M*_participated_ = 53.71, *SD* = 6.73).^1^

Considering the large difference in the number of male (47/200) versus female study participants, a sex difference test was conducted. Results showed that men had significantly higher levels of VO2AT (*F*_[1, 198]_ = 5.40, *p* = 0.021) and MCS scores at T3 (*F*_[1, 198]_ = 4.33, *p* = 0.039) than women, while there were no significant sex differences in the other variables (MCS_T1–T2, MCS_T3, PCS_T1–T4, SE_T1–T4) (*ps* > 0.005).

**Fig. 1:**
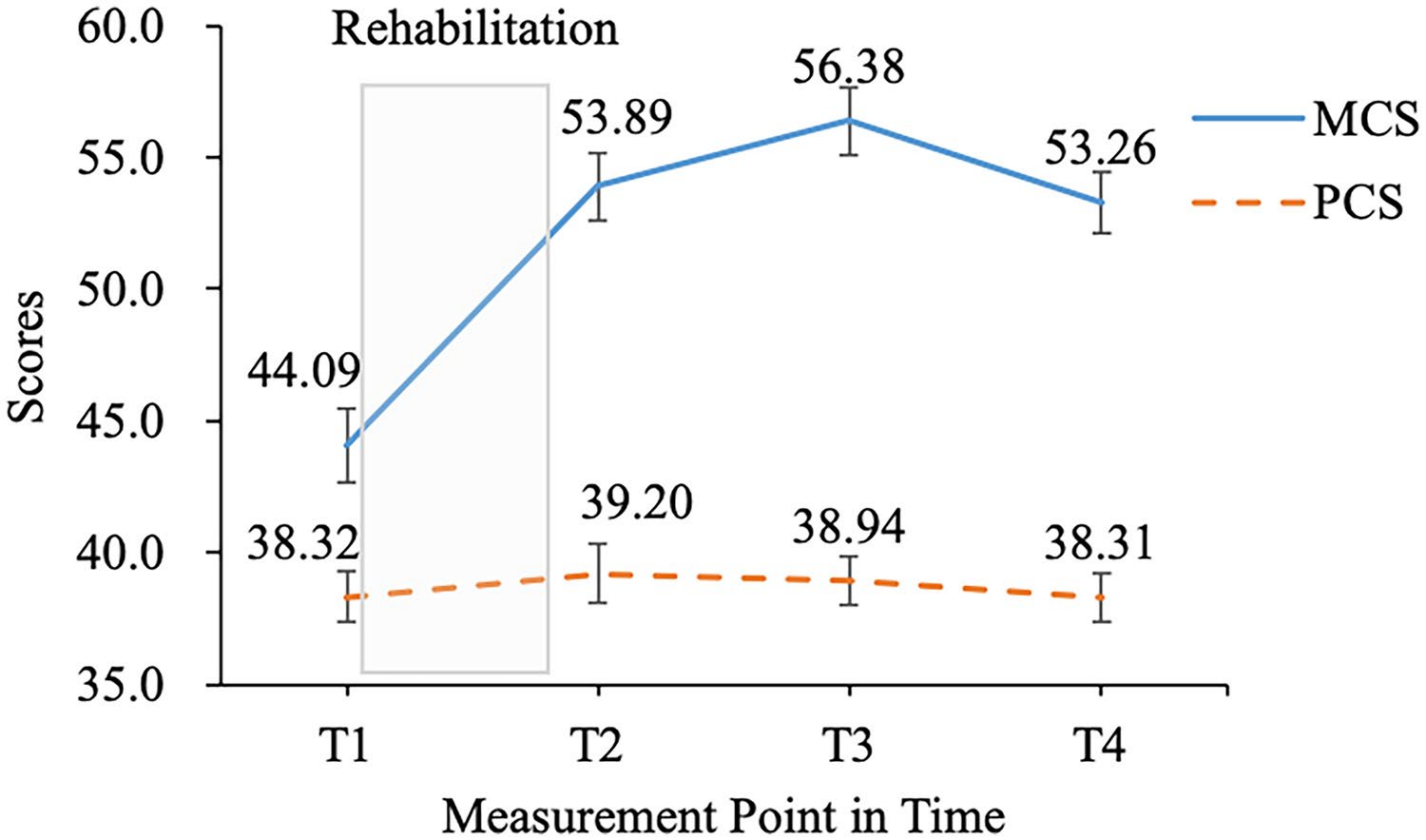
The developmental tendencies of mental and physical health. T1 ~ T4 = Time1 ~ Time4; the error bar is 95% confidence interval, and the gray box means the rehabilitation period; MCS, mental health component summary; PCS, physical health component summary

Simple descriptive statistical analysis was conducted for each variable, and the means, standard deviations, and partial correlation coefficients (with sex as the covariate) of each variable are shown in Table 3. The development tendencies of mental and physical health are depicted in Fig. 1. The results showed that VO2AT was related to mental health at T1 only, to physical health at T2–T4, and not related to self-efficacy.

## Repeated Measures ANCOVA of Means on Mental and Physical Health

The original dependent variables (VO2AT, MCS_T1–T4, PCS_T1–T4, and SE_T1–T4) deviated from the normal hypothesis (all *ps* < 0.025), and so, the normalization process was conducted for all dependent variables. A two-step normalization approach was used to transform continuous variables to normal [66], and the transformed data was used for the following ANCOVA and LGCM analyses.

An ANCOVA with sex as a covariate was conducted. Results showed the main effect of time for mental health was significant (*F*_[3, 196]_ = 5.25, *p* = 0.002, *η*^2^ = 0.026). Pairwise comparisons revealed the scores for mental health significantly increased from T1 to T3 (all *ps* < 0.011) and decreased between T3 and T4 (*p* < 0.001). However, mental health was at a higher level at T4 compared to T1 (*p* < 0.001). Furthermore, the mean scores for mental health showed a significant upward trend from T1 to T3, but a downward trend from T3 to T4 with the possibility of linear or quadratic changes. In contrast, the mean values of physical health were basically unchanged at each of the four measurements, with a non-significant main effect of time (*F*_[3, 196]_ = 2.10, *p* = 0.105, *η*_part_^2^ = 0.01) and non-significant pairwise comparisons (all *ps* > 0.050).

Moreover, self-efficacy also changed over time with a non-significant effect of time (*F*_[3, 196]_ = 3.05, *p* = 0.064, *η*_part_^2^ = 0.02), but all pairwise comparisons were significant (all *ps* < 0.001). The mean scores for self-efficacy significantly decreased from T1 to T2 but increased from T2 to T4. To investigate the dynamic change trend of participants’ mental and physical health over the four time points (hypotheses 1 and 2), models 1 and 2 were established below.^2^

### Model 1: Testing the Unconditional Linear LGCM for Mental Health Over Time

To explore whether the data was consistent with linear growth, model 1 was established. We set the loads of the slope for the first estimated linear unconditional LGCM at 0, 7, 12, and 15, based on the time points of the four measurements. As shown in Table 4, the linear model fit of the standardized result of the linear unconditional LGCM was poor [65]. The results showed that the intercept, or the initial mental health level of participants, was 47.48 (*p* < 0.001) and the slope was 0.55 (*p* < 0.001), which meant that mental health levels generally increased.

**Table 4:** The indicates of LGCM for model 1 to model 4

	AIC	BIC	X^2^(*df*)	RMSEA	95% CI for RMSEA	CFI	SRMR	Intercept	CI for intercept	Slope	CI for slope	Quadratic slope	CI for quadratic slope
Model 1: linear LGCM	5837.64	5867.32	84.90(5)	.28	[.23, .34]	.00	.17	47.48***	[14.55, 24.65]	.55***	[− 50.79, 13.49]	-	-
Model 2: quadratic LGCM	5764.19	5807.07	3.45(1)	.11	[.00, .25]	.94	.03	43.94***	[42.12, 45.75]	2.25***	[1.71, 2.79]	− .11***	[− .14, − .07]
Model 3: time-invariant LGCM	5764.50	5817.28	4.80(2)	.08	[.00, .19]	.93	.03	39.24***	[32.70, 45.77]	3.73***	[1.81, 5.66]	− .19***	[− .31, − .08]
Model 4: time-invariant and time-variant LGCM	5747.89	5813.86	18.06(14)	.04	[.00, .08]	.94	.05	37.06***	[30.13, 43.98]	4.43***	[2.52, 6.70]	− .31***	[− .49, − .13]

*LGCM* latent growth curve model, *RMSEA* root mean square error of approximation, *CFI* comparative fit index, *SRMR* standardized root mean square residual, *CI* 95% confidence intervals

^***^p < .001

### Model 2: Testing the Quadratic LGCM for Mental Health Over Time

To verify whether the data conform to the curve growth, model 2 was conducted. We again set the slope load at 0, 7, 12, and 15 in the hypothesized quadratic model (see Supplement 1) and set the quadratic load at 0, 49, 144, and 225. Model 2 fit the observed data better than model 1 (see Table 4), with a significant improvement in the model fit (_Δ_*χ*^2^(4) = 81.45, *p* < 0.001).

According to the standardized results of the quadratic unconditional model, the intercept was 43.94 (*p* < 0.001). Mental health showed an increasing trend during the four measurement points (slope = 2.25, *p* < 0.001). In addition, the mean of the slope decreased over time (quadratic slope =  − 0.11, *p* < 0.001), meaning that, during the four measurements, the mental health level increased from T1 to T3 (12 months after the beginning of rehabilitation) and then decreased from T3 to T4 (15 months after the beginning of rehabilitation).

While model 2 could describe the development tendency toward mental health, it could not reveal the reasons for the changes. To further explore the interrelating factors for mental health, we established models 3 and 4, which included the time-independent variable of VO2AT and the time-dependent variable of self-efficacy. In other words, whether changes occurred depending on other factors.

To verify whether the VO2AT and self-efficacy played a role in the trajectory of individuals’ mental health, the conditional LGCM was constructed in Models 3 and 4.

### Model 3: The Quadratic Conditional LGCM of Mental Health with Time-Invariant Covariates

Considering that sex may affect the model, the quadratic conditional LGCM was first run with sex as a covariate. The result showed that the LGCM with sex was nonconvergent, which means that participants’ sex did not have a predictive role in the quadratic model. Therefore, sex was not included in the subsequent quadratic models.

To explore whether initial physical fitness could predict the initial level and growth rate of mental health, model 3 added the VO2AT as the time-invariant covariate and the remaining settings were the same as model 2.

From the standardized results of the conditional time-invariant model, the intercept meant that the level of mental health at the beginning of rehabilitation was 39.24 (*p* < 0.001). The slope was 3.73 (*p* < 0.001), and the quadratic value was − 0.19 (*p* < 0.001), indicating that mental health showed an increasing trend but that this increasing trend decreased between the four time points (see Supplement 2).

The interrelation of VO2AT with mental health was significant on the slope (*γ*_β1_ =  − 0.096, *p* = 0.039) and the quadratic value (*γ*_β2_ = 0.01, *p* = 0.043) but was not significant on the intercept (*γ*_α_ = 0.30, *p* = 0.054). The result indicated that VO2AT played a negative role on slope, that was, the higher fitness capacity, the less the overall mental health growth. Besides, VO2AT played a positive role on the quadratic slope, meaning that individuals with higher fitness capacity had more stable mental health (larger VO2AT value contributed to larger negative quadratic value with the larger the opening of the quadratic curve, and the change of the mental health level was smaller). Moreover, VO2AT had no significant relationship with the intercept, which meant that the fitness capacity level of individuals played a subordinate role on the initial mental health level. Furthermore, oxygen reabsorption did not interrelate with the initial level of mental health.

### Model 4: The Quadratic Conditional LGCM of Mental Health with Time-Invariant and Time-Variant Covariates

**Fig. 2:**
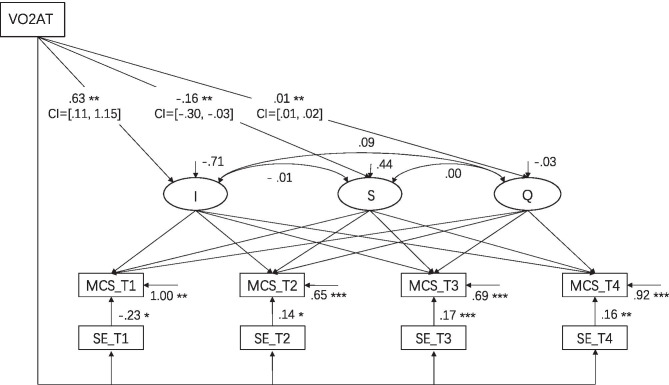
The standardized quadratic conditional LGCM of MCS with time-invariant and time-varying covariate (model 4). T1 ~ T4 = Time1 ~ Time4; LGCM, latent growth curve model; MCS, mental health component summary; SE, self-efficacy; I, intercept; S, slope; Q, quadratic slope; CI, 95% confidence intervals. *p < .050, **p < .010, ***p < .001

To test whether self-efficacy positively correlated with physical health at each time point, self-efficacy was added to model 4. Taking into consideration that self-efficacy was changing significantly over time and that this variable was measured four times, model 4 added self-efficacy as a time-variant covariate. The remaining settings were the same as model 3. The results are shown in Fig. 2, and the observed data fit the hypothesized model well, as shown in Table 4 [65].

From the standardized results of the conditional model, the intercept showed that the level of mental health at the beginning of the study was 37.06 (*p* < 0.001), the slope was 4.43 (*p* < 0.001), and the quadratic value was − 0.31 (*p* < 0.001), indicating that mental health showed an increasing trend but that this increasing trend decreased between the four time points.

The interrelation of VO2AT with mental health was significant on the intercept (*γ*_*α*_ = 0.63, *p* = 0.002), the slope (*γ*_*β*1_ =  − 0.15, *p* = 0.002), and the quadratic value (*γ*_*β*2_ = 0.01, *p* = 0.004). These results showed that individuals with a high fitness capacity had higher initial levels of mental health, a lower healthy mental growth rate, and a stable level of mental health (lager VO2AT contributed to larger negative quadratic value with bigger opening of quadratic curve).

In addition, the level of self-efficacy for health was negatively associated with the level of mental health at T1 (T1: *β* =  − 0.23, *p* = 0.010), while from T2 to T4 self-efficacy was positively related to mental health (T2: *β* = 0.14, *p* = 0.034; T3: *β* = 0.17, *p* < 0.001; T4: *β* = 0.16, *p* = 0.003), revealing the positive relationship between self-efficacy and mental health after rehabilitation treatment, and the potential effect of self-efficacy on the maintenance of mental health.

## Discussion

### Developmental Trajectories of Mental and Physical Health

The present study investigated the developmental trajectories of mental and physical health among medical rehabilitation patients in order to answer how mental and physical health changed over time and what roles physical fitness and self-efficacy played in these changes. The main finding of this study was that mental health underwent a nonlinear change, suggesting an increase during and after rehabilitation treatment and a decrease from the second follow-up time point (directly after rehabilitation), whereas physical health remained stable over time. Furthermore, rehabilitation and physical fitness played positive roles in the improvement of mental health, while self-efficacy did not.

Firstly, although the data did not fit the models very well, model 1 and model 2 showed that the quadratic model 2 was better than the linear model 1. Furthermore, model 3 verified hypothesis 1 with acceptable fittings of the results and revealed a U-shape development of mental health among medical rehabilitation patients. The quadratic model results indicate that rehabilitation treatment played a positive role in patients’ mental health. Mental health levels increased from the beginning of medical rehabilitation to the 12th month after the beginning of treatment. Although their mental health decreased during the 12th to the 15th month, it was still higher than it was initially. These results demonstrate the importance of medical rehabilitation and are consistent with previous studies [18, 67]. The results are also in line with Wienert, Schwarz [68], supporting the conclusion that work-related medical rehabilitation benefited the quality of life and mental health in cancer patients. Furthermore, the LGCM was used, and the results found that military veterans’ mental health demonstrated a quadratic change [69], and the workers’ psychological well-being showed linear changes [70]. It is possible that mental health is unstable and impressionable. Different measurements of time were used in this study, and in general, various interrelating factors like rehabilitation and target population might affect the shape of mental health’s developmental trajectory.

Furthermore, our ANOVA indicated that physical health status did not change, revealing that rehabilitation had little effect on patients’ physical health; therefore, further analysis was not conducted for the LGCM of physical health. These results are inconsistent with the hypotheses 2, 4, and 6, whose aims were to explore the change tendency and affecting factors for physical health. A possible reason may be that problems related to the patients’ health limited their access, frequency, and intensity of physical activity, and thus, the rehabilitation treatment may have not contributed significantly to a change in their physical health status, leading to the invariability of physical health.

### Role of Physical Fitness on the Developmental Trajectory of Mental Health

Consistent with hypothesis 3, that physical fitness would play a positive role in mental health, the results of model 3 showed that VO2AT/physical fitness predicted both the initial state and development tendency of mental health, revealing that patients with higher physical fitness had higher levels of mental health. The results also showed that physical capacity (VO2AT) was associated with the physical health component at T2–T4, but not at T1. The possible reason may be that, on the one hand, VO2AT could be a good indicator of subjective health trends over time but has limited relative value at the same time point. On the other hand, when patients have just entered rehabilitation, they may be far away from home and also not able to work, which can contribute to a negative mood and thereby one’s perception of pain, health, and quality of life; thus, they may report having poor health even though an objective assessment might indicate otherwise.

Previous studies have shown physical activity and physical conditions had a positive relationship with mental health [45, 71]. Furthermore, Ho, Louie [72] found physical activity improved adolescents’ mental health, and Aparicio, Marin-Jimenez [24] noted self-reported physical function was associated with mental health in perimenopausal women. In the current study, the better the participants’ level of physical fitness, the less their mental health changed during and after the rehabilitation treatment. This result showed that patients with high physical fitness had a more stable mental health status. In particular, after the rehabilitation treatment, the mental health of the individuals slowly declined and physical fitness played a buffering role in this debility. These results implicate the potency of physical fitness on mental health, especially among medical rehabilitation patients, and indicate that improving patients’ physical fitness could not only improve mental health during medical rehabilitation, but also alleviate the decline of mental health after rehabilitation treatment and therefore maintain mental health stability.

### The Interaction Between Physical Fitness and Self-Efficacy on the Developmental Trajectory of Mental Health

The fit between model 4 and the observed data was optimal, and therefore, it is consistent with hypothesis 5, which proposed that the interaction between physical fitness and self-efficacy would positively correlate with mental health after the rehabilitation treatment period. However, the results still suggest some implications: after the rehabilitation treatment, the long-term interrelations, with the rehabilitation treatment might deteriorate, and the patient’s mental health may slowly decline. In this situation, self-efficacy positively predicted the level of mental health, as higher self-efficacy led to better mental health. It is possible that, after the rehabilitation treatment, self-efficacy may have had a buffering effect on the decline of mental health. This result is consistent with previous studies [73–76] that found that self-efficacy was positively associated with mental health. One possible explanation is that self-efficacy always positively correlates with mental health, which is also described in the CCAM [35] and HAPA model [60, 77]. The potentially important positive role of self-efficacy on mental health was not shown at T1 and T2. This may be because patients were reminded of what they had learned and received support from the practitioners during the rehabilitation treatment, which masked the positive relationship between self-efficacy and mental health. However, after the rehabilitation treatment (T2-T4), patients would have to rely on their sense of self-efficacy; thus, the long-term association with rehabilitation treatment may wear off, and the true lasting effect of self-efficacy is reflected.

This result suggests that self-efficacy positively correlates with mental health after the rehabilitation treatment. More attention could be paid to patients with low self-efficacy because their mental health may decline faster than those with a higher level of self-efficacy after the rehabilitation treatment. Additionally, the improvement of their self-efficacy could also be considered a buffer for the mental health decline during the waning period of the rehabilitation treatment’s long-term interrelation.

### Theoretical and Practical Contributions

The finding that mental health is subject to quadratic growth, and that physical fitness and self-efficacy are associated with these changes have theoretical and practical implications. The present results broaden the theoretical understanding of the HAPA and CCAM models of lasting developmental trends in mental health in terms of the uniform pattern of mental and physical health with an increase and then a decrease and also show that both fitness level and self-efficacy matter for mental health. Past research about HAPA or CCAM has principally focused on health behavior [78–80], such as chronic illness [77], dietary behavior [81], physical activity [82], and Internet use [39]. This study extends the model into the realm of the developmental trajectory of mental health, demonstrating that orthopedic rehabilitation patients require support with building up a high fitness level and self-efficacy for reaching or maintaining good mental health. Few studies have demonstrated this link before, and future research should test this in an interventional design.

Moreover, previous studies on HAPA and CCAM used cross-sectional [23, 79] and longitudinal methods [80]. This study used four measurement points and conducted LGCM to extend the CCAM from the group level to the individual level, showing the development tendency of mental health was nonlinear (quadratic) and similar for all individuals. Specifically, with the combination of subjective and objective measurement methods, this study used the LGCM to demonstrate how to investigate the roles of physical fitness and self-efficacy at the initial level and the rate of change in mental and physical health during and after rehabilitation treatment. Having such a long-term perspective and offering critique and feedback on the developments, not only to the previous rehabilitation patients but also to clinics and funding agencies of such treatments, will open avenues for improving the effectiveness of such therapies.

Patients’ mental health decreased after the rehabilitation, which was also found in a previous study [83]. This confirms the assumption that a nonlinear developmental tendency may be a normal phenomenon for medical rehabilitation patients. A development trend like this may occur because rehabilitation patients’ may have chronic or reoccurring mental health symptoms [84, 85], which might be very salient to them. During their rehabilitation, it is therefore suggested that patients be encouraged not to focus on problematic work or life conditions, but instead strive for a health-promotion focus that contributes to their mental health [86]. For example, patients may need to be taught coping skills that can help them better manage a chronic illness, obtain support from their employer, and understand how to seek out or use one’s social support network. As such, future studies could investigate rehabilitation patients’ support network and coping skills for chronic and recurring mental and physical health issues with regard to development trends. Moreover, future research could examine the effects in more detail to identify how this could be done. The current research demonstrates the first methodological steps for doing so.

## Limitations and Future Research Directions

Several limitations of the study need to be noted. First, VO2AT was not measured at all time points. The data collection at T1 when VO2AT was measured occurred during the patient’s rehabilitation program. To receive meaningful data, VO2AT can only be measured in a standardized laboratory setting where the physical activity and the constraints of the participants can be monitored. Since the data collection at T2–T4 took place via CATI, it was not possible for us to measure VO2AT at these time points. Measurement would have required patients to return to the rehabilitation clinic at T2–T4, and we assumed that we would have a much greater drop-out rate. Furthermore, the measurement of VO2AT includes a 1-h spinning session with studied nurses, which is time-consuming, exhaustive for patients, and expensive. The level of physical fitness may also change over time; thus, future studies could consider measuring VO2AT at all time points. Second, self-efficacy was measured by only one item, which may lead to poor reliability; more measurements for self-efficacy, like the General Self-Efficacy Scale [87], should be considered in the future. Third, this study only used longitudinal measurements. In order to explore the role of rehabilitation treatment, a control group and an experimental design would be interesting in future studies to shed more light on specific intervention variables and how they influence related health outcomes. Fourth, it is unknown whether the findings of this study could be applied to other populations, such as workers with job burnout [88], unemployed persons [89, 90], or military personnel [69, 91]. Further research could generalize the conclusions among other populations to explore the internal mechanisms of the mental and physical changes and lay a theoretical foundation for further intervention research, such as interventions for occupational health [92]. Fifth, this study assessed the patient’s status at 7, 12, and 15 months after leaving the hospital without structured maintenance treatment, and most patients only receive long-term treatment by a primary care therapist and physician [93]. Thus, considering the concrete treatment patients receive and its effect on long-term development of patients’ mental and physical health as well as their actual ability to work and return to work would be needed. It would also be interesting to explore which combinations of therapy are most helpful for rehabilitation patients to regain full functionality. Last, only the role of self-efficacy in mental health was investigated in this study; other important social-cognitive variables, such as social support [23, 94], merit further exploration.

## Conclusion

In conclusion, this study revealed a quadratic growth in mental health among medical rehabilitation patients. The quadratic model results indicate that, at the individual level, the rehabilitation treatment played a positive role in each medical rehabilitation patients’ mental health and had a long-term interrelation. In addition, their physical health maintained a stable state over time. The initial level of physical fitness was positively associated with the initial level of mental health, as well as the stability of the development tendency. Self-efficacy positively correlated with the level of mental health after the rehabilitation treatment.

Based on the results of this study, some recommendations are put forth for practitioners. First, because physical fitness is beneficial to mental health in medical rehabilitation patients, more attention should be given to physical capacity in rehabilitation for the specific benefit of improving and maintaining patients’ mental health. Second, in order to help rehabilitation patients to return to work sooner, social-cognitive determinants like self-management training or the training of work-directed self-efficacy should be considered. Our findings suggest that such interventions should be flexible and time-specific because participants develop over time. Self-management training might be very appropriate in this role as it has been shown that rehabilitation patients profit from such interventions, which include developing coping strategies, increasing knowledge of one’s disease, learning and implementing self-management behaviors, and promoting physical activity [95, 96]. Additionally, because such interventions transfer knowledge and techniques to overcome physical and psychological impairments, it would be possible to design such interventions with a stepwise approach that takes the development of patients over time into account.

## Supplementary Information

Below is the link to the electronic supplementary material.

Supplementary file1 (DOCX 19 KB)

## Footnotes

1 Information about the raw data can be obtained from the PI of the project.

2 To understand sensitivity to missing data, the LGCMs based on data without the imputation procedure were conducted (see Supplement 3). To normalize the data without the imputation procedure, the two-step approach was used. The results showed that Model 1 did not fit the data well because the SRMR was .16, which is higher than the recommended value of .08. Models 2 to 4 did not converge. It is possible that the amount of data without imputation was insufficient (for the data at T4, there were only 73 valid data points).

## Declarations

### Research Involving Human and Animal Participants

This article does not contain any studies with animals performed by any of the authors.
