# Supplementary material for: Health Status Stability of Patients in a Medical Rehabilitation Program: What Are the Roles of Time, Physical Fitness Level, and Self-efficacy?
Source: Int J Behav Med. 2021 Dec 23;29(5):624–37. doi: 10.1007/s12529-021-10046-6 (PMC9525393; doi:10.1007/s12529-021-10046-6)
Supplement: Supplementary file 1 — Supplementary file1 (DOCX 19 KB) [file 12529_2021_10046_MOESM1_ESM.docx]

Supplementary Information

**Supplement**

***Supplement 1.*** The standardized quadratic unconditional LGCM of MCS (Model 2)

*Note*. T1~T4 = Time1 ~ Time4; LGCM = Latent Growth Curve Model, MCS = Mental Health Component Summary; I = Intercept, S = Slope, Q = Quadratic slope; ** *p* < .010 *** *p* < .001.

*Supplement 2.* The quadratic conditional LGCM of MCS with time-invariant covariates (Model 3)

*Note*. T1~T4 = Time1 ~ Time4; LGCM = Latent Growth Curve Model, MCS = Mental Health Component Summary; I = Intercept, S = Slope, Q = Quadratic slope; CI = 95% Confidence Intervals.

* *p* < .050; ** *p* < .010, *** *p* < .001.

| ***Supplement 3*.** | | | | | | | | | | | | | |
| --- | --- | --- | --- | --- | --- | --- | --- | --- | --- | --- | --- | --- | --- |
| *The indicates of LGCM for Model 1 to Model 4 based on the data without the imputation after the two-step normalization* | | | | | | | | | | | | | |
|  | AIC | BIC | *χ*^^2^ (*df*) | RMSEA | 95%CI for RMSEA | CFI | SRMR | Intercept | CI for Intercept | Slope | CI for Slope | Quadratic slope | CI for Quadratic slope |
| Model 1: Linear LGCM | 1105.66 | 1135.11 | .71(5) | .00 | [.000, .000] | 1.00 | 0.17 | 50.00*** | [49.87, 50.13] | .00 | [-.02, .02] | - | - |
| Model 2: Quadratic LGCM | The model did not convergence | | | | | | | | | | | | |
| Model 3: Time-invariant LGCM | The model did not convergence | | | | | | | | | | | | |
| Model 4: Time-invariant and time-variant LGCM | The model did not convergence | | | | | | | | | | | | |
| *Note*. LGCM = Latent Growth Curve Model; RMSEA = Root Mean Square Error of Approximation, CFI = Comparative Fit Index, SRMR = Standardized Root Mean Square Residual; CI = 95% Confidence Intervals. **** p < .001* | | | | | | | | | | | | | |
